# Giant cardiac schwannoma around the left atrium: a case report

**DOI:** 10.1093/jscr/rjae738

**Published:** 2024-11-27

**Authors:** Ryohei Ushioda, Boonsap Sakboon, Dit Yoongtong, Jaroen Cheewinmethasiri, Hiroyuki Kamiya, Nuttapon Arayawudhikul

**Affiliations:** Cardiovascular and Thoracic Unit, Department of Surgery, Lampang Hospital, Lampang 52000, Thailand; Department of Cardiac Surgery, Asahikawa Medical University, Asahikawa, Hokkaido 078-8510, Japan; Cardiovascular and Thoracic Unit, Department of Surgery, Lampang Hospital, Lampang 52000, Thailand; Cardiovascular and Thoracic Unit, Department of Surgery, Lampang Hospital, Lampang 52000, Thailand; Cardiovascular and Thoracic Unit, Department of Surgery, Lampang Hospital, Lampang 52000, Thailand; Department of Cardiac Surgery, Asahikawa Medical University, Asahikawa, Hokkaido 078-8510, Japan; Cardiovascular and Thoracic Unit, Department of Surgery, Lampang Hospital, Lampang 52000, Thailand

**Keywords:** cardiac schwannoma, left thoracotomy, cardiopulmonary bypass

## Abstract

A 57-year-old male presented with dyspnea and an enlarged cardiac silhouette on a chest X-ray. Further evaluation with contrast-enhanced computed tomography revealed a giant heterogeneous mediastinal mass, ~8.9 × 7.3 × 12.2 cm, with peripheral calcifications. Surgical resection was performed via a left thoracotomy approach using the left fifth intercostal space. Cardiopulmonary bypass was established through the femoral vessels for safer and more controlled resection. The tumor, contiguous with the left atrium, was successfully excised using two Endo GIA staplers. Pathological examination confirmed the diagnosis of schwannoma. This case demonstrates that the left thoracotomy approach with cardiopulmonary bypass and the use of Endo GIA staplers is a feasible and effective option for resecting large, well-defined cardiac schwannomas.

## Introduction

Cardiac schwannomas are rare, typically benign tumors that develop in the Schwann cells surrounding the heart [1, 2]. However, they still cause problems if they grow large enough to compress nearby structures or interfere with the heart’s normal functioning [3]. In this case, the patient presented with dyspnea due to a giant cardiac schwannoma compressing surrounding structures, necessitating surgical resection. Here, we report the successful resection of the tumor around the left atrium using a left thoracotomy.

## Case report

**Figure 1:**
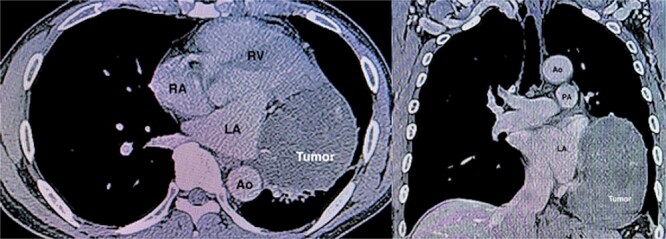
CT angiography demonstrating a huge tumor (8.9 × 7.3 × 12.2 cm) measuring adjacent to the left atrium (LA) and left ventricle (LV). Ao, ascending aorta; RA, right atrium; RV, right ventricle; PA, pulmonary artery.

A 57-year-old male patient presented with dyspnea. His medical history, physical examination, and laboratory tests were unremarkable. The electrocardiogram revealed regular sinus rhythm without ST changes. A chest X-ray detected an abnormal shadow in the right lung hilum. Transthoracic echocardiography (TTE) revealed an extracardiac mass with a halo measuring 5.5 × 7.5 cm attached to the left atrial posterior wall. Contrast-enhanced computed tomography (CT) revealed a heterogeneously enhancing 8.9 × 7.3 × 12.2 cm mediastinal mass with peripheral calcifications silhouetting the left cardiac border with fat plane separation (Fig. 1). The exact kind of the tumor, including whether benign or malignant, was unknown preoperatively. We planned to remove the tumor both for diagnostic purposes and treatment. We didn’t perform a preoperative pathological diagnosis in this case due to the risks of cardiac injury and bleeding. Surgical resection via left thoracotomy was performed, as the patient expressed a desire for an early return to daily life and work, as this approach avoids a median sternotomy and reduces the risk of complications, such as mediastinitis.

**Figure 2:**
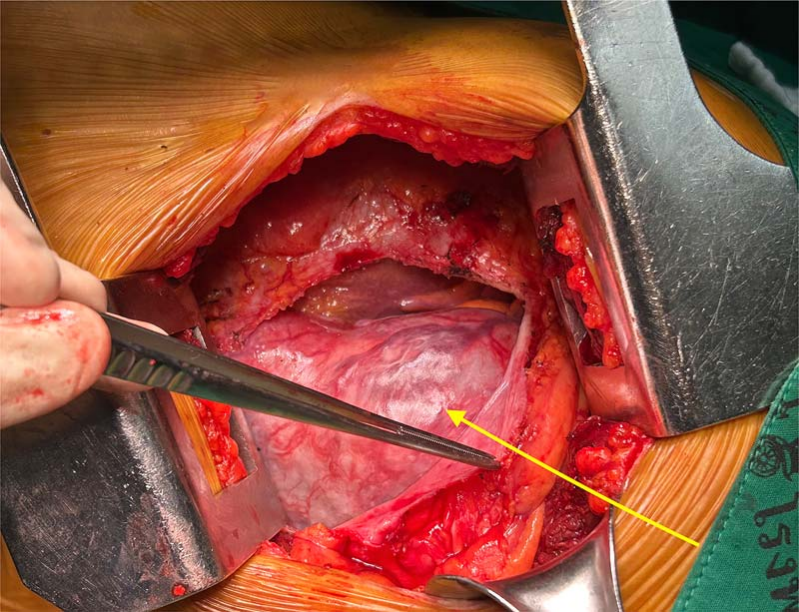
Operative findings. The schwannoma (arrow) was seen from the left thoracotomy when opening the pericardium.

**Figure 3:**
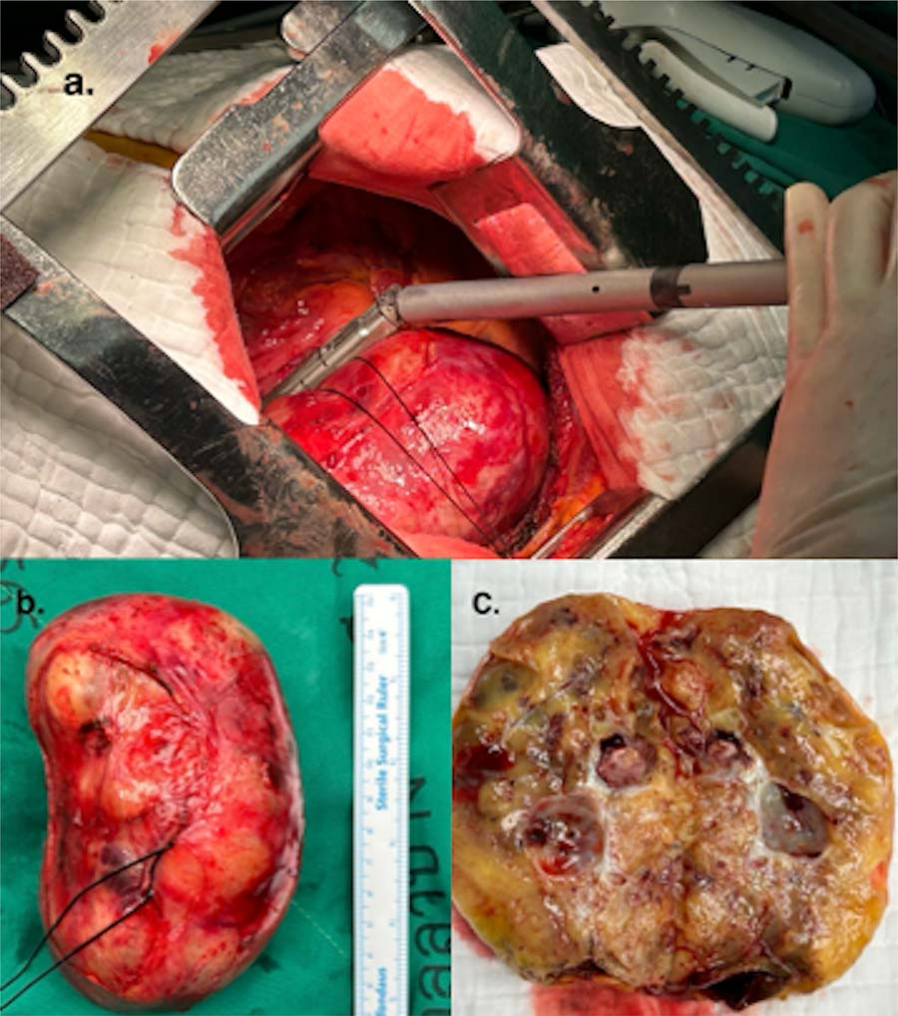
(a) Resected schwannoma with an Endo GIA stapler. (b) Resected giant cardiac schwannoma (12 × 7 cm). (c) Macro findings of the tumor showing a cystic structure with wall thickening.

**Figure 4:**
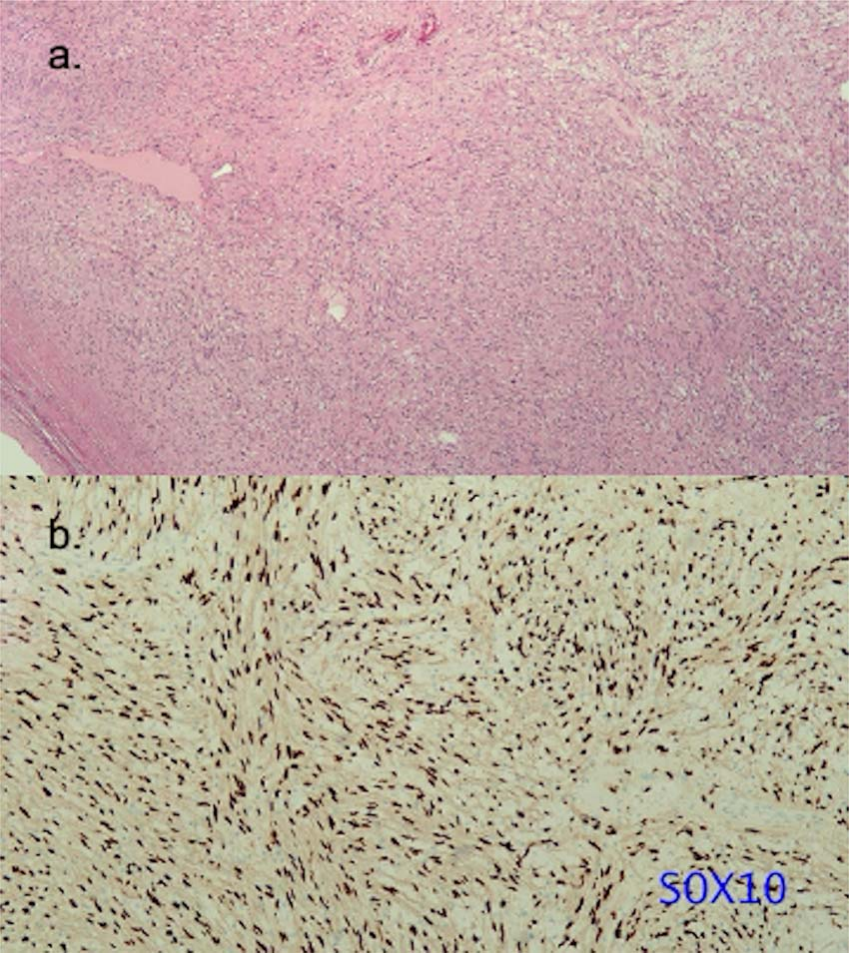
Histopathology of the resected schwannoma. (a) Histologic analysis of the specimen revealing alternative cell-rich Antoni A type area and cell-poor Antoni B type area (hematoxylin and eosin stain). The resected specimen did not show pathological continuity between the left atrium and the tumor. (b) Tumor cells exhibiting intense positive staining for S-100 protein.

The patient was positioned by elevating the left chest at 30°–40° and intubated with a double-lumen endotracheal tube. Thoracotomy was established in the left fifth intercostal space. The mass was exposed after the pericardial opening, and it appeared contiguous with the left atrium (Fig. 2). Given the tumor’s size and location, achieving a safe resection was challenging, prompting the decision to use cardiopulmonary bypass (CPB) for a more controlled and secure procedure. CPB was established with the femoral artery and femoral vein. The mass was easily resected with a total of two Endo GIA staplers 60–2.0 mm after conversion to CPB (Fig. 3). The procedure was performed under on-pump beating, with a total CPB time of 42 min. The total operative time was 2 h and 36 min. The estimated blood loss was 300 ml, and no blood transfusion was required during the surgery. The postoperative course was uneventful, and the patient was discharged 3 days postoperatively. Microscopic examination revealed a mixed proliferation of Antoni type A and Antoni type B tissue. The tumor cells demonstrated positive staining for S-100 protein. Based on these findings (Fig. 4), the pathology report confirmed the diagnosis of schwannoma.

## Discussion

Schwannoma in primary neurogenic tumors of the cardiac is an extremely rare disease. Cardiac schwannomas account for only 0.2%–2% of all primary cardiac tumors [4]. Most of these schwannomas appear on the right side of the heart, close to the interatrial septum. The location may originate from cardiac branches of the vagus nerve and cardiac plexus [5, 6]. The clinical manifestations of schwannomas include various symptoms, including dyspnea on exertion, chest pain, shortness of breath, palpitations, and arrhythmias by compression and obstruction. Contrarily, >30% of patients demonstrated no related symptoms [7], such as in this case.

In cardiac schwannoma, TTE, CT, and magnetic resonance imaging (MRI) can help to determine the location and extent of the mass and the involvement of other structures [25]. Tumors are mostly heterogeneous masses with cystic changes, hemorrhages, and calcifications. Uneven and mild enhancement may even occur. Some lesions have a broad base and shallow lobes, and most lesions have a clear boundary. The fibrous capsule is also one of the identification points of schwannomas from other tumors [7, 8]. Additionally, MRI can aid in differentiating benign schwannomas from malignant peripheral nerve sheath tumors, providing valuable diagnostic information [9].

Cardiac schwannomas exhibit a favorable prognosis, and surgery is the primary treatment. A meta-analysis of primary cardiac schwannoma case reports [10] concluded surgery as the primary treatment option for both benign and malignant cases and was the only factor related to a relative improvement in survival. The 3-year postoperative survival rate was 100% with no recurrence in benign cases. In the present case, the tumor was resected using the Endo GIA stapler. However, there are no established reports or evidence supporting the use of staplers for cardiac tumor resection. Therefore, long-term follow-up is essential to ensure early detection of any complications, like a recurrence.

The operative decision to use CPB and approach depends on several factors, including tumor location, size, and extent and the patient’s clinical condition and comorbidities. Approximately 30 reports described ‘schwannoma’ or ‘neurilemomas’, as seen in PubMed. Of these, this report included 21 resection cases using CPB (Table 1). This is the first report of tumor resection using the left thoracotomy approach. Previous reports indicated the median sternotomy as the incision of choice because patching the defect after tumor resection is frequently necessary. On the other hand, patch repair was performed in five cases, not associated with the size or location of the tumor. We successfully used a thoracotomy approach to resect a pedunculated tumor larger than 10 cm located in front of the heart. While the thoracotomy approach has the advantage of avoiding mediastinitis, it is technically difficult to do the operation due to limited working space. The thoracotomy can be performed regardless of tumor size when using CPB, particularly if the tumor is lobulated and located in front of the heart. Conversely, median sternotomy and cardiac arrest with CPB should be favored for tumors with unclear borders, high degrees of myocardial infiltration, or locations on the posterior side of the heart.

**Table 1:** Case of surgery for schwannoma with a cardiopulmonary bypass.

No.	Author	Year	Age	Sex	Location of tumor	size (cm)	Surgical approach	Surgical method	Aortic clamp
1	Betancourt *et al.* [12]	1979	32	F	Intracavitary tumor attached to parietal band of crista	8.7 × 6.25	Median sternotomy	Resection	N/A
2	Forbes *et al.* [13]	1994	35	M	Posterior LA between inferior PV and CS	4 × 7	Median sternotomy	Resection, LA patch	+
3	Hashimoto *et al.* [6]	1998	46	F	Between SVC and ascending Ao	12 × 8 × 7	Median sternotomy	Resection	N/A
4	Bizzarri *et al.* [8]	2001	72	M	Intracavitary tumor attached to floor of RA, close to AV groove	5 × 4 × 4	Median sternotomy	Resection	N/A
5	Sirlak *et al.* [14]	2003	61	F	LA	9.5 × 8.5 × 6.5	Median sternotomy	Resection	N/A
6	Nakamura *et al.* [5]	2003	33	F	Anterior RA extending LA and PV	5 × 5.2 × 4.5	Median sternotomy	Resection, PV, and LA patch	+
7	Jassal *et al.* [15]	2003	49	F	RA adjacent to the AV groove	6.4 × 5.5 × 3.4	Median sternotomy	Resection	N/A
8	Xiao-Dong *et al.* [16]	2005	51	F	RA	10.2 × 10	Median sternotomy	Resection	N/A
9	Stolf *et al.* [17]	2006	56	F	RA, close to the cavo-atrial junction	6.0 × 4.8	Median sternotomy	Resection, RA patch	−
10	Sevimli *et al.* [18]	2007	57	F	The free wall of the LV	5.5 × 6	Median sternotomy	Resection	N/A
11	La Francesca *et al.* [19]	2007	30	F	Anterior and lateral surface of the superior half of the LV	4 × 4 × 9	Median sternotomy	Resection, CABG	N/A
12	Early *et al.* [20]	2007	57	M	Posterolateral wall of the RA extending to the interatrial septum	4.3 × 5.2	Median sternotomy	Resection	N/A
13	Anderson *et al.* [21]	2011	67	M	RA involving the interatrial septum	3.1 × 2.5 × 1.7	Median sternotomy	Resection, AVR	+
14	Elstner *et al.* [22]	2013	65	M	Lateral wall of the LPA	5.2 × 4.5 × 4.1	Median sternotomy	Resection, CABG, PA patch	N/A
15	Hwang *et al.* [23]	2014	55	F	LA, attached to the left atrial appendage	4.3 × 4 × 3	Median sternotomy	Resection	+
16	Son *et al.* [9]	2015	42	F	Atrial roof between the aorta and the SVC	10 × 9.5	Median sternotomy	Resection	+
17	Huang *et al.* [24]	2020	53	M	Behind the ascending Ao	8.2 × 7.1 × 6.9	Median sternotomy	Resection	N/A
18	Yokoyama *et al.* [25]	2021	46	M	Posterior wall of the LA	1.4 × 1.6	Median sternotomy	Resection, atrial septal patch	+
19	Wang *et al.* [7]	2021	64	F	Anterior RV	2.8 × 2.0	N/A	Resection	N/A
20	Li *et al.* [26]	2021	70	F	Posterior pericardium	4.7 × 5.9 × 8.1	N/A	Resection, patch	N/A
21	Present case	2023	57	M	Attached to the LA, close to AV groove	8.9 × 7.3 × 12.2	Left thoracotomy	Resection	−

M, male; F, female; N/A, not available; LA, left atrium; PV, pulmonary vein; CS, coronary sinus; SVC, superior vena cava; Ao, aorta; RA, right atrium; AV, atrioventricular; LV, left ventricle; CABG, coronary artery bypass surgery; AVR, aortic valve replacement; PA, pulmonary artery; RV, right ventricle.

The left thoracotomy approach with CPB can be a feasible option for well-defined tumors located in front of the heart. The Endo GIA stapler is useful in cases with limited working space, as demonstrated in this case. However, long-term follow-up is essential to monitor.
